# Magnetic resonance anisotropy in CeB_6_: an entangled state of the art

**DOI:** 10.1038/srep39196

**Published:** 2016-12-16

**Authors:** A. V. Semeno, M. I. Gilmanov, A. V. Bogach, V. N. Krasnorussky, A. N. Samarin, N. A. Samarin, N. E. Sluchanko, N. Yu. Shitsevalova, V. B. Filipov, V. V. Glushkov, S. V. Demishev

**Affiliations:** 1 Moscow Institute of Physics and Technology, 9 Institutsky lane, Dolgoprudny 141700 Moscow region, Russia; 2 Prokhorov General Physics Institute of RAS, Vavilov street, 38, 119991 Moscow, Russia; 3 Institute for Problems of Materials Science of NASU, 3, Krzhyzhanovsky Str., Kiev 03680, Ukraine; 4 National Research University Higher School of Economics, Myasnitskaya street, 20, 101000 Moscow, Russia

## Abstract

Electron spin resonance (ESR) in strongly correlated metals is an exciting phenomenon, as strong spin fluctuations in this class of materials broaden extremely the absorption line below the detection limit. In this respect, ESR observation in CeB_6_ provides a unique chance to inspect Ce^3+^ magnetic state in the antiferroquadrupole (AFQ) phase. We apply the original high frequency (60 GHz) experimental technique to extract the temperature and angular dependences of *g*-factor, line width and oscillating magnetization. Experimental data show unambiguously that the modern ESR theory in the AFQ phase considering the Γ_8_ ground state of Ce^3+^ ion completely fails to predict both the *g*-factor magnitude and its angular dependence. Alignment of the external magnetic field along [100] axis induces a strong (more than twofold) broadening of ESR line width with respect to the other crystallographic directions and results also in the anomalous temperature dependences of the *g*-factor and oscillating magnetization. In this experimental geometry the latter parameter surprisingly exceeds total static magnetization by 20% at T* ~ 2.5 K. We argue that the unusual physical picture of ESR in CeB_6_ may be strongly affected by spin fluctuations and dynamic collective effects predominantly pronounced in [100] direction.

Magnetic resonance in strongly correlated metals is an exciting phenomenon, as long as if any, it is observed in very unfavorable conditions. Indeed, spin fluctuations in this class of materials are strong enough and broaden usually magnetic resonance line width to practically unobservable values. For example, in YbRh_2_Si_2_ the estimate of the spin fluctuations contribution to the line width *W* gives *W* ~ 37 T, although narrow electron spin resonance (ESR) was detected in this material with the help of X-band spectrometer at resonant field about 0.2 T^1^. This discrepancy stimulated an intense search for the physical mechanism, which might lead to narrowing of the ESR line width to observable values. According to existing theories, resonant line broadening by spin fluctuations may be overcame by ferromagnetic (FM) correlations^2^,^3^,^4^. On a qualitative level, this result is nothing but the second advent of well known Korringa mechanism of spin relaxation, where ESR line width is inversely proportional to magnetic susceptibility *W* ~ 1/χ^5^. Indeed, FM correlations enhance χ and thus reduce *W*.

This clear physical picture is blurred by the recent discovery of magnetic resonance in cerium hexaboride CeB_6_^6^,^7^. This material is known to be driven by antiferromagnetic (AFM) interactions rather than FM ones^8^. Additional difficulty arises from the complicated character of the magnetic phase diagram of CeB_6_, consisting of the paramagnetic (P) phase, so-called antiferroquadrupole (AFQ) phase, where ordering of the Ce f-orbitals is expected, and complex antiferromagnetic (AF) phase^8^. It turned out that ESR in CeB_6_ detected in AFQ phase was missing in P phase and, therefore, it was supposed that this phenomenon may be somehow caused by orbital ordering effects^6^. However, subsequent studies have revealed that resonance magnetic oscillations in CeB_6_ are caused by oscillating magnetization *M*_0_, which is less than total static sample magnetization *M*^7^. Moreover, the temperature dependence of the oscillating part at the resonance field *M*_0_(*B*_*res*_, *T*) is different from *M(B*_*res*_, *T*), and demonstrate clear FM behavior *M*_0_(*B*_*res*_, *T*) ~ (*T* − *Θ*_CW_)^−1^ with *Θ*_CW_ ~ 2 K^7^. Therefore ESR in CeB_6_ indicates non-trivial magnetization structure and existence of an oscillating FM component in the AF system. From one hand, this observation confirmed general character of the ESR mechanism in a strongly correlated system with strong spin fluctuations^2^,^3^,^4^. On the other hand, it was considered as an argument against orbital ordering model in CeB_6_^7^, because the possibility of FM correlations was not foreseen in available models of the AFQ phase at the moment of ESR discovery. It is worth noting that the FM component in CeB_6_ was later confirmed in neutron scattering experiments^9^,^10^. Moreover, recent comparative study revealed an excellent agreement between the dispersion laws *ω(B*) for the main gapless resonant mode in the AFQ phase subtracted from the ESR measurements and from the neutron scattering in magnetic field data^11^. This mode corresponding to the *g*-factor *g* ~ 1.6–1.7 may be traced up to ω/2π ~ 350 GHz for magnetic field aligned along [110] crystallographic direction^11^,^12^. However, the situation with the high-frequency ESR experiments in CeB_6_ is more complicated as long as for ω/2π > 200 GHz the second mode with the *g*-factor *g* ~ 1.2–1.3 is observed simultaneously with the main ESR mode^11^,^12^.

Unique physics of magnetic resonance in CeB_6_ allowed calling it “exception to exceptions”^11^,^13^ and stimulated development of the corresponding theory^11^,^12^,^13^,^14^. Assuming the Γ_8_ ground state of Ce^3+^ ion, Schlottmann suggested that the related effects of orbital ordering may result in the onset of FM correlations in AFQ phase^13^,^14^, which meet the experimental situation^7^,^9^,^10^,^11^. As long as four ESR modes exist for the Γ_8_ quartet^13^,^14^ some physical mechanism is required to reduce the number of observed magnetic excitations. According to^13^,^14^ the AFQ order quenches some transitions so that the only one magnetic resonance may be observed. An attempt to explain doubling of the ESR line consisted in hypothesis that high-energy microwave quantum may somehow destroy the AFQ phase^14^ but under this assumption it is difficult to understand either the perfect conservation of main resonant mode^11^,^12^, or well established enhancement of stability of the AFQ phase in high magnetic field corresponding to ω/2π > 200 GHz^8^. In this respect, some revisiting of the applicability of the model^14^ for interpretation of the high-frequency ESR measurements^11^,^12^ may be required.

Nevertheless, when relatively low ESR frequency region is considered, the theory makes some quantitative predictions to be verified by experiment. Namely, the angular dependence of the *g*-factor corresponding to the single resonance was computed for the Γ_8_ ground state in the AFQ phase^13^,^14^. The anisotropy effects in ESR in CeB_6_ have not been investigated up to now, and all the available information is limited to the case when the magnetic field is aligned along [110] crystal axis^6^,^7^,^11^,^12^. In this paper, we aim to fill this gap intending to make a detailed inspection of the predictions of the theoretical model suggested in refs 13 and 14. An original experimental technique of cavity measurements, which allows finding the full set of ESR spectroscopic parameters including the oscillating magnetization, line width and *g*-factor in the case of strongly correlated metals^7^,^15^,^16^ is applied. This investigation may shed more light on the nature of unusual static and dynamic magnetic properties of CeB_6_, which still remain the subject of debates in spite of more than a forty years old history of studies of this amazing material.

## Results

### Temperature dependences of ESR characteristics along main crystal axes

**Figure 1:**
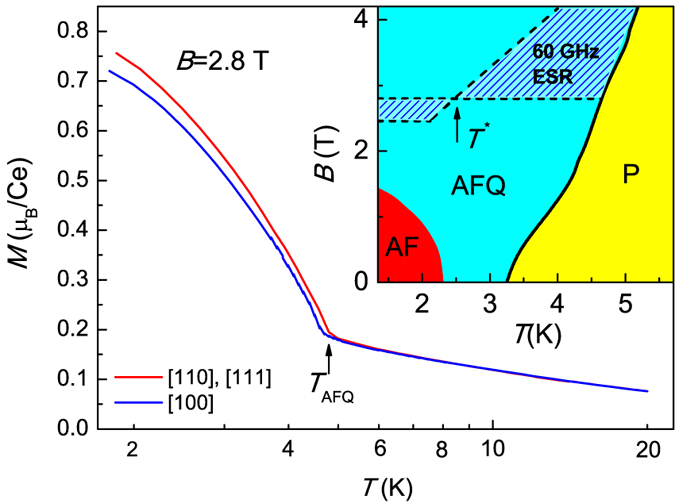
Temperature dependences of static magnetization in magnetic field *B* = 2.8 T applied along different crystallographic directions (main panel). Inset presents the magnetic phase diagram of CeB_6_. The B-T domain where 60 GHz electron spin resonance may be observed is shaded. The anisotropic phase boundary for the AF phase is shown for **B** ‖ [110] direction.

Before we start analysis of the ESR data, it is instructive to consider static magnetization data and ESR area on the *B-T* magnetic phase diagram (Fig. 1). In zero magnetic field lowering of temperature results in the sequence of phase transitions: P phase to AFQ phase at *T*_AFQ_ = 3.2 K and AFQ phase to AF phase at *T*_AF_ = 2.3 K. In magnetic field the transition temperature *T*_AFQ_(*B*) increases, whereas *T*_AF_(*B*) decreases (inset in Fig. 1). It is worth noting that the P-AFQ phase boundary is almost isotropic while the transition into the AF phase depends strongly on the crystallographic direction. Our 60 GHz ESR measurements show that the resonant fields *B*_*res*_(*T*) depend also on the direction of magnetic field with respect to crystal axes covering wide shaded area located inside AFQ phase (inset in Fig. 1). This observation is unusual as long as the temperature dependences of total static magnetization *M* (*B* = const, *T*) in fixed magnetic field do not demonstrate strong anisotropy (see main panel of Fig. 1, which presents the data for *B* = 2.8 T corresponding to the horizontal boundaries limiting ESR area on the magnetic phase diagram).

**Figure 2:**
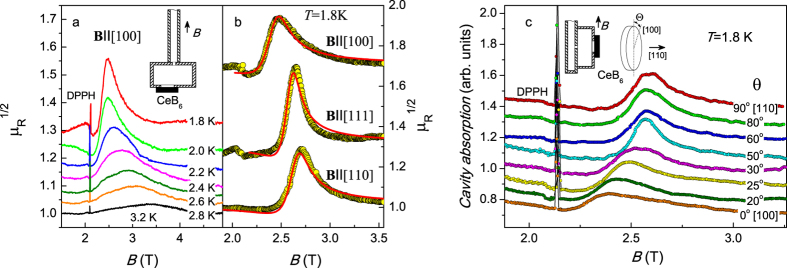
ESR spectra (60 GHz) calibrated in units of magnetic permeability μ_R_ for **B** ‖ [100] at different temperatures (panel a) and examples of the approximation of the ESR line shape at *T* = 1.8 K for different crystallographic directions (panel b; points- experiment, solid lines- fits in the model of localized magnetic moments). Some μ_R_(*B*) curves are shifted for clarity from the level μ_R_ = 1. Panel c represents field dependences of the ESR spectra for various sample orientation at *T* = 1.8 K. DPPH marks the reference signal of diphenyl picryl hydrazyl. Insets in the panels a and c display the corresponding experimental geometry.

The ESR data processing schema^7^,^15^,^16^ allowed obtaining resonant magnetoabsorption spectra in the units of magnetic permeability μ_R_. As a general rule, the ESR line broadens when the temperature is approaching to the P-AFQ phase boundary (Fig. 2a). For **B** ‖ [110] and **B** ‖ [111] the resonant field depends weakly on temperature in contrast to the case of **B** ‖ [100], where the resonant field increases by ~1.4 times under the temperature variation from 1.8 K to 3.2 K (Fig. 2a).

**Figure 3:**
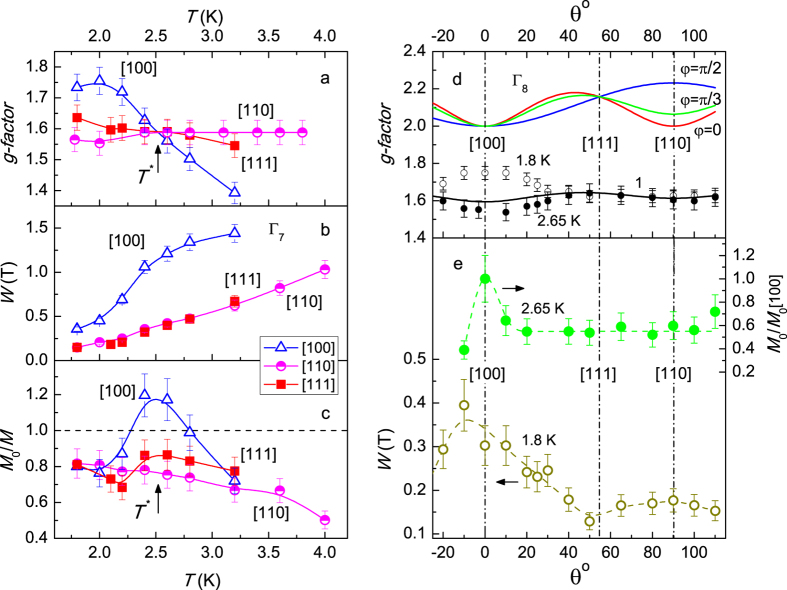
(**a–c**) Temperature dependences of spectroscopic parameters for different crystallographic directions (see text for details). Angular dependences of (**d**) *g*-factor at different temperatures and (**e**) reduced oscillating magnetization *M*_0_ at *T* = 2.65 K and line width *W* at *T* = 1.8 K. Points with error bars denote experimental data. Solid line 1 in the panel d denotes the fit by empirical relation (3) (see text for details). Lines in the panels a-c and e are guide to the eye.

The examples of approximation of the ESR line shape in the localized magnetic moments (LMM) model^7^,^15^ are shown in Fig. 2b. It is visible that theoretical analysis may adequately reproduce experimental data. Similar fits were performed for all the temperatures studied and were used to obtain temperature dependences for the *g*-factors *g(T*), line widths *W(T*) and oscillating magnetization *M*_0_(*T*) as described in refs 7,15,16. The results are presented in Fig. 3a–c. Temperature variation of the *g*-factor is weak for **B** ‖ [110] and **B** ‖ [111] and this parameter is about *g* ≈ 1.6 in agreement with the data reported previously^7^ (Fig. 3a). The dependence *g(T*) in the case **B** ‖ [100] is noticeably different. For this experimental geometry *g* ~ 1.4 at *T* = 3.2 K and lowering of temperature results in an increase of the *g*-factor up to the values *g* ~ 1.7–1.75 for *T* < 2.2 K (Fig. 3a). It is worth noting that the *g*-factors for all main crystallographic directions coincide at *T* = T* ~ 2.5 K and *g* [110], *g* [111] > *g* [100] in the range *T* > T* and *g* [110], *g* [111] < *g* [100] for *T* < T* (Fig. 3a). This observation may indicate that magnetic state of CeB_6_ in the ESR resonant field may change at T*. This opportunity will be considered below in more details.

The selected character of [100] direction is clearly demonstrated by the temperature dependences of the ESR line width (Fig. 3b). The *W(T*) curves for **B** ‖ [110] and **B** ‖ [111] coincide within the experimental error, whereas the external magnetic field aligned along [100] direction results in the enhancement of this parameter by the factor of ∼2 (Fig. 3b). Moreover the *W(T*) dependence for **B** ‖ [100] tends to saturate when the temperature approaches P – AFQ phase boundary in contrast to the cases **B** ‖ [110] and **B** ‖ [111] (Fig. 3b). Interesting that characteristic temperature T*, which follows from the *g*-factors temperature dependences, does not appear in *W(T*) data (Fig. 3a,b).

The most striking feature is observed in the case of oscillating magnetization *M*_0_ (Fig. 3c). As long as this parameter in CeB_6_ may differ from total static magnetization *M*^7^, we have analyzed ratio *M*_0_/*M*. First of all, the inequality *M*_0_/*M* < 1 holds at any temperature studied for **B** ‖ [110] and the temperature dependence of the reduced oscillating magnetization is smooth. This behavior is in fair agreement with the results of the previous study^7^. In the case **B** ‖ [111] the same condition *M*_0_/*M* < 1 is valid, but temperature dependence *M*_0_/*M* = *f(T*) is different (Fig. 3c). In the vicinity of T*T* lowering temperature results in decrease of *M*_0_(*T*) and hence of the ratio *M*_0_/*M* as long as temperature dependence of the total static magnetization does not show any peculiarities around *T* ~ T* ~ 2.5 K (Fig. 1). The considered feature of the *M*_0_/*M* = *f(T*) becomes more pronounced in the experimental geometry **B** ‖ [100]. The ratio *M*_0_/*M* reaches a maximum at *T* ≈ T* and then drops by 1.5 times in the range *T* < T* (Fig. 3c). It is amazing that the region *T* ~ T* for **B** ‖ [100] is characterized by anomalous magnitude of oscillating magnetization, which *exceeds* total static magnetization (*M*_0_/*M* > 1, Fig. 3c). This experimental finding is far from ordinary common sense expectations and, to the best of our knowledge, has never been reported in any ESR studies.

### Angular dependences of the ESR spectra

The experimental scheme for the measurement of the ESR angular dependences is shown in inset in Fig. 2c. The axis of the cylindrical cavity was parallel to [110] direction (inset in Fig. 2c). Thus, the external field **B** passes through all principal crystallographic directions [100], [110] and [111] under sample rotation. The signal from the reference 2,2-diphenyl-1-picrylhydrazyl (DPPH) sample was used to control the variation of microwave field magnitude in the cavity. In these measurements, the procedure of absolute calibration in units of magnetic permeability becomes difficult, although line shape analysis suitable for determination of the *g*-factor and the line width is still possible. Therefore below we will report only relative variation of the oscillating magnetization *M*_0_.

The example of the angular dependence of the ESR spectra is presented in Fig. 2c. Both the position of the resonance and the width of the resonant magnetoabsorption line depends on the angle *θ*, which is measured from the axis [100]. Angular dependences of the *g*-factor *g(θ*) obtained at temperatures 1.8 K (*T* < T*) and 2.65 K (*T* > T*) are weak and coincide to each other except the region Δ*θ* = ±30° around [100] direction (Fig. 3d). In this temperature-angle domain lowering temperature results in noticeable growth of the *g*-factor up to ~13% (Fig. 3d).

It is worth noting that the selected character of the [100] direction develops clearly not only in the *g*-factor, but also in other ESR parameters. The normalized oscillating magnetization *M*_0_/*M*_0_([100]) = *f(θ*) jumps just around the field direction **B** ‖ [100] for *T* = 2.65 K ~ T* (upper curve in Fig. 3e) and the line width *W(θ*) broadening is also related with this specific crystallographic direction (lower curve in Fig. 3e). Note that the magnitude of the *M*_0_(*θ*) jump and *W(θ*) enhancement at *θ* = 0 (**B** ‖ [100]) agree reasonably with the data obtained in temperature measurements along different axes (panels b-c and e in Fig. 3). Some discrepancies may be attributed to a small misalignment of the sample in different ESR setups and to more complicated background in the experiment with the rotating cavity.

## Discussion

The starting point for the analysis of the ESR parameters is to compare the obtained *g(θ*) data with the predictions of the ESR theory for CeB_6_^13^,^14^, where the orbital ordering in the AFQ phase is considered assuming the Γ_8_ ground state of Ce^3+^ ion. The calculation for the Γ_8_ state, which symmetry allows quadrupole and octupole moments^8^, gives^13^,^14^


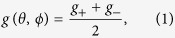


where


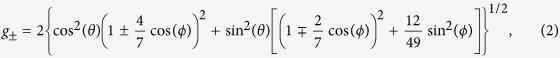


Here the angle *φ* is a free parameter of the model^13^,^14^, which fixes spatial orientation of the 4*f* orbitals in the AFQ phase^13^,^14^. Equations (1) and (2) suggest that *g(θ*) should lie within the band min {*g(θ*, 0), *g(θ, π*/2} ≤ *g(θ*) ≤ max{*g(θ*, 0), *g(θ, π*/2} with the corresponding functions *g(θ*, 0) and *g(θ, π*/2) plotted in Fig. 3d. It is remarkable that this estimation fails completely to describe the observed *g*-factor magnitude. Indeed, the expected *g*-factor value is located between 2 and 2.23, whereas experiment gives g ~ 1.4–1.75 (Fig. 3a,d). Moreover, as long as theoretical expressions (1)–(2) do not depend directly on temperature, the only way for introducing of the *g*-factor temperature dependence is an assumption concerning temperature dependence of the angle *φ* = *φ* (*T*). However, the strongest temperature variation occurs along [100] direction, for which equations (1)–(2) give *g* ≡ 2 to be independent on the *φ* value (Fig. 3d). Therefore the existing theory based on the Γ_8_ ground state assumption is unable to account for either observed absolute values of the *g*-factor, or the anomalous temperature dependence of the *g*-factor along [100] axis. Nevertheless, the qualitative coupling mechanism, which reduces the four ESR transitions to a single resonant feature in the AFQ phase^13^,^14^, seems to be valid in the studied frequency range.

The fact that [100] axis in CeB_6_ is unique in static magnetic properties of the AFQ and AF phases is well known and is believed to be attributed to specific spatial orientation of 4f orbitals. Our results demonstrate an anomalous character of this direction in dynamic magnetic properties as well. However, several important questions arise: (1) Why does ESR theory based on orbital ordering model in the Γ_8_ ground state^13^,^14^ fail to describe the experimental data? (2) What is the reason for anomalous temperature dependence of the *g*-factor, line width and oscillating magnetization when the external magnetic field is applied along [100]? (3) What kind of magnetic transition may occur at T*? (4) How may oscillating magnetization exceed static magnetization? We believe that these questions should be addressed to the improved theory of ESR in strongly correlated metals covering the case of CeB_6_, which is beyond the scope of the present experimental work. Nevertheless, to shed more light on the entangled state of the art for ESR in CeB_6_, we will qualitatively comment these challenges below.

**Figure 4:**
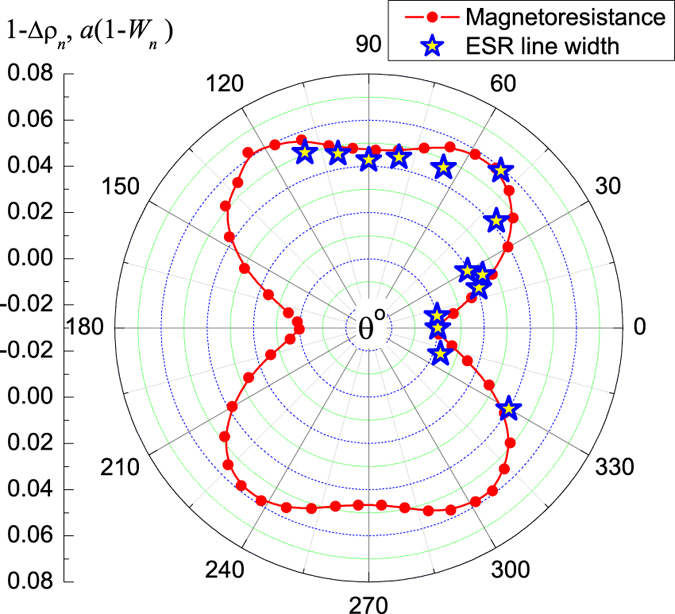
Correlation between angular dependences of the magnetoresistance and line width at *T* = 1.8 K. Magnetoresistance data correspond to *B* = 2.8 T.

1. The existing ESR theory^13^,^14^ may not take into account additional physical effects, which may renormalize *g*-factor value. For example, the exchange coupling of Ce moments to conducting electrons may alter the position of the resonance and hence the observed *g*-factor (similar to Knight-shift effect). The theoretical equations (1) and (2), which describe *g* (*θ, φ*), may meet experimental *g*_*exp*_(*θ, φ*) data if the following empirical relation is assumed
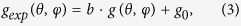
where *b* ≈ 0.3, *g*_0_ ≈ 1 and φ = π/3 (see line 1 in Fig. 3d). This formula describes well the angular dependence of the *g*-factor except the anomalous region around ***B*** ‖ [100] direction (Fig. 3d). The above estimate shows that the effect of reduction of the *g*-factor calculated for the Γ_8_ ground state in refs 13 and 14 is strong, and therefore dominating AF type of coupling is the must. However, this supposition is in disagreement with the existing theory of magnetic resonance in strongly correlated metals^2^,^3^,^4^,^13^,^14^, where FM correlations are responsible for the feasibility of the magnetic resonance. In CeB_6_ the P-phase is indeed Kondo-like one and is driven by AF correlations, but the discussed magnetic resonance is *not* observed just in this region of the magnetic phase diagram in agreement with the theoretical predictions^2^,^3^,^4^,^13^,^14^. Moreover, it is well established by ESR and neutron scattering data^7^,^9^,^11^ that oscillating contribution to magnetization in CeB_6_ is driven by FM rather than AF correlations. In addition, the influence of AF correlation is well understood in the ESR physics at least in the mean-field approximation^17^,^18^,^19^. For example, this type of magnetic interactions causes an increase of the ESR line width with lowering temperature^17^,^18^,^19^, which does not meet the experiment, where opposite behavior of *W(T*) is observed (Fig. 3b). Consequently the above reason for the *g*-factor change seems to be highly debatable. However, AF correlations in CeB_6_ may be responsible for some other effects seen in dynamic magnetic properties (see below).
2. Now let us discuss anomalous temperature dependences of the ESR characteristics. First of all, the ESR line broadens when the magnetic field is applied along [100] direction (Fig. 3b,e). It is known that the ESR line width in various strongly correlated materials may be controlled by spin fluctuations^1^,^2^,^3^,^4^,^20^ so it is possible to suppose that the same mechanism works in CeB_6_. Spin fluctuations may also affect magnetic scattering in CeB_6_, which is damped by applied magnetic field leading to negative magnetoresistance. Therefore it is interesting to compare *W(θ*) data (Fig. 3e) with the angular dependences of the magnetoresistance Δρ(*B, θ*) = ρ(*B, θ*) − ρ(*B* = 0) at the same temperature *T* = 1.8 K in the field *B* ~ 2.8 T corresponding to ESR region on the magnetic phase diagram. We found that magnetoresistance Δρ_*n*_ = Δρ(*B, θ*)/Δρ(*B*, 0) and line width *W*_*n*_ = *W(θ*)/*W*(0) normalized to the corresponding values for **B** ‖ [100] direction may be linked in a simple way 1 − Δρ_*n*_ = *a*(1 − *W*_*n*_) where *a* ~ 0.1 is numerical coefficient (Fig. 4). Nice correlation between the Δρ(*θ*) and *W(θ*) dependences confirms the spin fluctuations role in the line broadening. This result is in agreement with the neutron scattering experiments by Portnichenko *et al*.^11^, where magnon mode is more intense (and hence less damped) in **B** ‖ [110] direction, rather than in **B** ‖ [001] direction. Thus it may be concluded that spin fluctuations in CeB_6_ are strongly anisotropic and reach maximal magnitude for **B** ‖ [100]. As long as in CeB_6_ spin fluctuations are expected to be associated with the itinerant component of the total magnetization^21^, they will also change the local field inside the sample, which in turn will lead to the enhancement of the observed *g*-factor value just for **B** ‖ [100] (Fig. 3a,d) due to FM coupling^7^,^9^.
3. In the case when temperature dependences of oscillating magnetization (Fig. 3) are taken *per se*, it is possible to say that at *T* ~ T* the magnetization decreases for **B** ‖ [100] and **B** ‖ [111] so that the sample undergoes an anisotropic AF transition. The problem is that this hypothetical transition is observed in the AFQ phase, which is different from the AF phase at any temperature, and appears in dynamic magnetic properties governed by FM correlations but not in static ones (see Fig. 1). Recent investigation shows a strong coupling of the AF and AFQ order parameters in zero magnetic field^9^ therefore it is not excluded that some AF fluctuations may exist above *T*_AF_(*B*) for finite magnetic field inside the AFQ phase. If these fluctuations are viewed at the short-time scale corresponding to ESR magnetic oscillations 2π/*ω* ~ 1.7 · 10^−11^ s and time of life for fluctuations is high enough, the dynamical picture will resemble simple disordered antiferromagnet and the transition at *T* ~ T* may be some kind of virtual spin-glass transition. Apparently, AF spin fluctuations are averaged in static magnetic properties being unable to produce any observable AF order as visible from Fig. 1.
4. Now let us analyze possible reasons for anomalous excessive oscillating magnetization along [100] axis (Fig. 3). The hint for the interpretation of this unusual effect may be found in the Abrahams-Wölfle theory, where magnetic resonance in the limits of Kondo impurity and Kondo lattice was considered^2^. In both limits, the ESR is a collective many-body effect due to strong coupling between itinerant electrons and LMM of f-electrons^2^. As long as expression for the dynamic susceptibility *χ(ω*) for the concentrated system (Kondo lattice) was obtained under simplified model assumptions relevant to the case of YbRh_2_Si_2_ rather than to the case of CeB_6_, we will make below an estimate using general results for the Kondo impurity^2^. In the latter case, dynamic susceptibility acquires the form

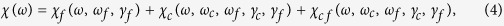


where the cross-term *χ*_*cf*_ describing interaction effects develops in addition to the contributions of localized electrons *χ*_*f*_ and itinerant electrons *χ*_*c*_. The parameters *ω*_*f*_ = *g*_*f*_ · *μ*_*B*_ · *B, ω*_*c*_ = *g*_*c*_ · *μ*_*B*_ · *B* and *γ*_*f*_, *γ*_*c*_ denote the ESR frequencies and unperturbed ESR line widths for LMM and band electrons, respectively^2^. For *ω* = 0 cross-term turns to zero *χ*_*cf*_ = 0 and static susceptibility is the sum of the localized electrons susceptibility *χ*_*f*_ and the Pauli-like term *χ*_*c*_^2^. In the case *ω*_*f*_ > *γ*_*f*_ and |*ω*_*f*_ − *ω*_*c*_| < < *γ*_*c*_ the expressions obtained in ref. 2 suggest a resonance condition *ω* = *ω*_*f*_, and at this frequency *χ*_*f*_ equals total static susceptibility of LMM system, whereas *χ*_*c*_ = 0. At the same time the cross-term *χ*_*cf*_ does not vanish at *ω* = *ω*_*f*_ and gives rise to excessive resonant contribution, which is missing in static data. The straightforward mapping of the *χ(ω*) in the case *ω*_*f*_ > *γ*_*f*_ and |*ω*_*f*_ − *ω*_*c*_| < < *γ*_*c*_ to the standard model of some oscillating LMM^7^,^22^ used in our data analysis results in the simple estimate of excessive dynamic magnetization Δ*M*_0_ ~ 2*M*_0_ · *g*_*c*_ · *γ*_*f*_ /[*g*_*f*_ · *γ*_*c*_(1 + *χ*_*c*_(*ω* = 0)/*χ*_*f*_(*ω* = *0*))] caused by the interaction of the itinerant and localized electrons, whereas the observed ESR line width *W* coincides well with the parameter *γ*_*f*_. As long as *g*-factors *g*_*f*_. *g*_*c*_ together with the line widths *γ*_*f*_, γc and partial susceptibilities *χ*_*f*_, *χ*_*c*_ are unknown functions of temperature, any quantitative estimates for the case of CeB_6_ are not possible. Nevertheless, it may be concluded that any increase of *γ*_*f*_ (i.e. of the ESR line width) will increase oscillating magnetization so that the condition *M*_0_ > *M* is not forbidden in this model. This supposition agrees with the experimental data, where both enhancement of *W* and anomalous growth of *M*_0_ correspond to [100] direction (Fig. 3). However, the Kondo impurity paradigm^2^ may be considered only as a starting point for accounting of the many-body effects in the concentrated limit and more theoretical work is required for the explanation of the observed oscillating magnetization anomaly.

Summarizing up, the study of the ESR anisotropy in the AFQ phase of CeB_6_ performed in the present work confirms strong reputation of this material as “exception to exceptions”, which provides a challenge to the models of the AFQ phase based on orbital ordering effects in the Γ_8_ ground state. Theoretical predictions for the *g*-factor value and its angular dependence deviate strongly from experiment. The physical picture of magnetic resonance seems to be essentially influenced by spin fluctuations having the strongest amplitude along [100] crystallographic axis. These spin fluctuations may be responsible for the strong broadening of the ESR line width and may cause an anomalous temperature dependence of the *g*-factor and oscillating magnetization *M*_0_ when the magnetic field is aligned around [100] direction. Surprisingly, the latter parameter exceeds total static magnetization *M*, which may be a consequence of a specific interaction between itinerant and localized electrons. Clarifying of the entangled physical picture of ESR in CeB_6_ may be important for development of the adequate rigorous theory of magnetism in this exciting material and may give a new impulse for research of ESR in strongly correlated metals.

## Methods

ESR measurements are performed at high quality single crystals identical to those studied in refs 6 and 7. Details concerning samples preparation can be found elsewhere^9^. Description of the ESR cavity spectrometer is given in ref. 15. The use of Agilent PNA network analyzer for generation and detection of microwave radiation results in enhancement of the signal-to-noise ratio by the factor of 10 with respect to ESR experiments carried out earlier in refs 6 and 7. The network analyzer allows performing continuous resonant frequency correction (peak following) that eliminates any time and field-dependent distortions of the ESR line shape. Sample temperature is stabilized by Cryotel 1.5/300 temperature controller with a 10^−4^ relative measurement accuracy and up to 5 mK low temperature stability in the range *T* < 60 K.

The ESR technique used includes special geometry of cavity measurements allowing the absolute calibration of microwave absorption by the metallic sample in units of magnetic permeability^7^,^15^,^16^. In this geometry, the cavity bottom is made of thin copper foil with a small hole at the maximum of microwave magnetic field. The measured CeB_6_ crystal is mounted outside the cavity in a way to cover the hole. For good electrical contact the conductive silver paint is used to fix the sample to the foil. Therefore, only central part of the measured sample is accessible to the microwave field and provides the ESR response, so any distortion of the line shape caused by magnetic field inhomogeneity due to demagnetization effect is eliminated^7^,^15^,^16^. ESR line shape analysis performed in ref. 7 unambiguously showed that ESR in CeB_6_ corresponds to resonant magnetic oscillations of some LMM. In this situation the application of the absolute calibration procedure described in detail in refs 7, 15 and 16 and subsequent model approximation of the line shape allows obtaining the full set of spectroscopic parameters, including *g*-factor, line width *W* and oscillating magnetization *M*_0_. It is worth noting that the determination of the latter parameter is difficult in standard ESR experiments especially in metals, so that information about *M*_0_ is missing as a rule. The details of the calculation procedure are provided in ref. 15.

In the present work, the ESR is measured with the help of cylindrical cavities operating at 60 GHz TE_011_ mode. Two types of the cavity experimental schemes are used. In the first case, the axis of the cavity is parallel to external magnetic field **B** and orientation of the sample is fixed with respect to **B** and microwave field. This geometry is used to measure temperature dependences of the ESR parameters, when the magnetic field is parallel to principal crystallographic directions [100], [110] and [111]. In the second case, vector **B** is directed perpendicular to the cavity axis and our installation allowed rotation of the cavity with the sample. In this experiment, it is possible to measure detail angular dependence of the ESR line with respect to the external magnetic field. It is necessary to emphasize the distinction of the resonance conditions in the above two geometries which is substantial for further data analysis. Due to gyrotropic origin of ESR the effective resonant function of magnetic permeability *μ*_*eff*_ (*B*) includes two elements of permeability tensor *μ(B*) and *μ*_*α*_(*B*)^23^. While in the case **k** ‖ **B** ESR response is defined by two circularly polarized functions *μ*_*±*_ = *μ*±*μ*_*α*_ the scheme with **k** ⊥ **B** results in 
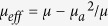
23. The difference in the resonance line position is strong for materials with large microwave magnetic susceptibility |*χ*_*1*_, *χ*_*2*_| ∼ 1 and remains remarkable in CeB_6_ at the lowest experimental temperatures (∼2% at *T* = 1.8 K). So we put special attention on the consistence of the experimental data taken in two setups.

In order to make chosen ESR technique feasible, the measurements of the microwave cavity absorption should be added by the magnetization and magnetoresistance measurements as explained in refs 7, 15 and 16. The DC magnetoresistance is measured by the four-probe technique at home-made installation described in ref. 24. Magnetic measurements up to 5 T have been carried out with the help of SQUID magnetometer MPMS-5 (Quantum Design).

## Additional Information

**How to cite this article:** Semeno, A. V. *et al*. Magnetic resonance anisotropy in CeB_6_: an entangled state of the art. *Sci. Rep.* **6**, 39196; doi: 10.1038/srep39196 (2016).

**Publisher's note:** Springer Nature remains neutral with regard to jurisdictional claims in published maps and institutional affiliations.
